# Evaluation of the Efficacy and Safety of a Marine-Derived *Bacillus* Strain for Use as an In-Feed Probiotic for Newly Weaned Pigs

**DOI:** 10.1371/journal.pone.0088599

**Published:** 2014-02-26

**Authors:** Maria Luz Prieto, Laurie O'Sullivan, Shiau Pin Tan, Peter McLoughlin, Helen Hughes, Orla O'Donovan, Mary C. Rea, Robert M. Kent, Joseph P. Cassidy, Gillian E. Gardiner, Peadar G. Lawlor

**Affiliations:** 1 Eco-Innovation Research Centre, Department of Chemical and Life Sciences, Waterford Institute of Technology, Waterford, Ireland; 2 Teagasc, Food Research Centre, Moorepark, Fermoy, Co. Cork, Ireland; 3 Veterinary Sciences Centre, University College Dublin, Belfield, Dublin, Ireland; 4 Pig Development Department, Animal and Grassland Research and Innovation Centre, Teagasc, Moorepark, Fermoy, Co. Cork, Ireland; 5 Alimentary Pharmabiotic Centre, University College Cork, Cork, Ireland; German Institute of Human Nutrition Potsdam-Rehbrücke, Germany

## Abstract

Forty eight individual pigs (8.7±0.26 kg) weaned at 28±1 d of age were used in a 22-d study to evaluate the effect of oral administration of a *Bacillus pumilus* spore suspension on growth performance and health indicators. Treatments (*n* = 16) were: (1) non-medicated diet; (2) medicated diet with apramycin (200 mg/kg) and pharmacological levels of zinc oxide (2,500 mg zinc/kg) and (3) *B. pumilus* diet (non-medicated diet + 10^10^ spores/day *B. pumilus*). Final body weight and average daily gain tended to be lower (*P* = 0.07) and feed conversion ratio was worsened (*P*<0.05) for the medicated treatment compared to the *B. pumilus* treatment. Ileal *E. coli* counts were lower for the *B. pumilus* and medicated treatments compared to the non-medicated treatment (*P*<0.05), perhaps as a result of increased ileal propionic acid concentrations (*P*<0.001). However, the medicated treatment reduced fecal (*P*<0.001) and cecal (*P*<0.05) *Lactobacillus* counts and tended to reduce the total cecal short chain fatty acid (SCFA) concentration (*P* = 0.10). Liver weights were lighter and concentrations of liver enzymes higher (*P*<0.05) in pigs on the medicated treatment compared to those on the non-medicated or *B. pumilus* treatments. Pigs on the *B. pumilus* treatment had lower overall lymphocyte and higher granulocyte percentages (*P*<0.001) and higher numbers of jejunal goblet cells (*P*<0.01) than pigs on either of the other two treatments or the non-medicated treatment, respectively. However, histopathological examination of the small intestine, kidneys and liver revealed no abnormalities. Overall, the *B. pumilus* treatment decreased ileal *E. coli* counts in a manner similar to the medicated treatment but without the adverse effects on growth performance, *Lactobacillus* counts, cecal SCFA concentration and possible liver toxicity experienced with the medicated treatment.

## Introduction

Weaning is a stressful event for young pigs characterized by gastrointestinal disturbances caused by physiological, immunological and microbiological changes within the gastrointestinal tract (GIT) [1]. During this period, pigs are highly susceptible to enteric diseases, and those caused by *Escherichia coli* (e.g. post-weaning diarrhea and edema disease) are responsible for considerable economic losses in the pig industry [1], [2]. As a result, in-feed antibiotics have long been used for the elimination or reduction of pathogenic bacteria, in particular *E. coli*, during the post-weaning period [1]. However, the routine use of in-feed antibiotics was banned in the EU in 2006 (Regulation EC/1831/2003;[3]), although their use is still permitted under veterinary prescription as the need arises. For instance, apramycin (Apralan G200, Elanco Animal Health, Eli Lilly & Co. Ltd) was prescribed for use on the pig farm where the current study was conducted to control persistent edema disease during the post-weaning period. However, antibiotic-resistance is a major human health issue and effective alternatives to antibiotics are required [1]. In-feed zinc oxide, at pharmacological concentrations (i.e. concentrations in excess of normal dietary requirements) is also commonly used for enteric disease prevention in weaned pigs but there are concerns about its accumulation in the environment [1].

Probiotics are defined as ‘live microorganisms which when administered in adequate amounts confer a health benefit on the host’ [4]. They offer potential as an alternative to antibiotics for pigs, both as a means of controlling enteric pathogens and improving growth rate and feed conversion [1], [5]. Together with modulation of the immune system and competitive exclusion, antimicrobial production is one of the suggested mechanisms of action of probiotics [6]. The latter can therefore be considered a probiotic trait and is often listed as one of the properties required of a strain for it to be considered probiotic [7]. This is backed up by the fact that strains selected *in vitro* for their anti-*E. coli* activity, have proven successful in reducing *E. coli* shedding, preventing diarrhea and improving growth performance in pigs [8]–[13]. However, as is usually the case with probiotics, benefits are strain-specific [5].

The marine environment is largely untapped as a source of probiotics but should not be overlooked given that it is a potential source of novel microorganisms and that antimicrobial production is common amongst marine microflora [14]. In fact, *in vitro* data from our group have demonstrated that marine bacteria have potential as probiotics for animal production [15], [16]. One strain of *Bacillus pumilus* showed most promise as it satisfies a number of probiotic criteria and has activity against *E. coli* without being cytotoxic. However, as with any potential feed additive, observations made *in vitro* need to be substantiated with *in vivo* data and to date, this marine *B. pumilus* strain has not been tested *in vivo*.

The objective of the present study was therefore to evaluate this pre-screened *Bacillus* strain for use as an in-feed probiotic for newly weaned pigs in comparison to a negative control treatment without antibiotic or pharmacological levels of zinc oxide (non-medicated treatment) and a positive control treatment containing apramycin and pharmacological levels of zinc oxide (medicated treatment). Key parameters including growth performance and health indicators were investigated in order to evaluate safety and efficacy *in vivo*.

## Materials and Methods

### Ethical Approval

The pig study complied with European Union Council Directive 91/630/EEC which lays down minimum standards for the protection of pigs and European Union Council Directive 98/58/EC which concerns the protection of animals kept for farming purposes. Ethical approval was obtained from the Waterford Institute of Technology ethics committee and an experimental license (number B100/4229) was obtained from the Irish Department of Health and Children.

### Animals and experimental design

This study was conducted on the pig unit at the Teagasc, Pig Development Department, Moorepark, Fermoy, Co. Cork, Ireland, which at the time was experiencing edema disease during the post-weaning period. Forty-eight crossbred (Large White × Landrace) pigs (24 male and 24 female; 8.7±0.26 kg) were weaned at 28±1 d of age and blocked by sex, weight and litter origin, before being randomly assigned as individual pigs to one of three dietary treatments (*n* = 16 pigs/treatment) as follows: (1) non-medicated diet (negative control); (2) medicated diet containing 200 mg apramycin/kg (Apralan G200, Elanco Animal Health, Eli Lilly & Co., Basingstoke, Hampshire, UK) and zinc oxide (2,500 mg zinc /kg provided from Zincotec, Provimi Ltd., Lichfield, Staffordshire, UK) (positive control); and (3) non-medicated diet + ∼10^10^ spores *B. pumilus* WIT 588 daily (prepared and administered as outlined below). Treatments were administered continuously for 22 d post-weaning and pigs were provided with *ad libitum* access to feed and water.

The dietary and chemical composition of the experimental diets is presented in Table 1. The diets were manufactured in the Moorepark feed mill and were formulated to meet or exceed the National Research Council requirements for weaned pigs [17]. All phase 1 diets were formulated to 16.2 MJ/kg digestible energy and 16.2 g/kg lysine using the same ingredients except that apramycin and pharmacological levels of zinc oxide were added to the medicated diet. Similarly, all phase 2 diets were formulated to 15.0 MJ/kg digestible energy and 15.0 g/kg lysine using the same ingredients except that apramycin and pharmacological levels of zinc oxide were added to the medicated diet. All diets were fed in 3 mm pellet form. Phase 1 diets were fed for 1 week after which phase 2 diets were fed for the remainder of the experiment.

**Table 1:** Ingredient composition and nutrient content of experimental diets (on an air dry basis).

Diet type	Phase 1		Phase 2	
	Non-medicated^1^	Medicated	Non-medicated^1^	Medicated
Ingredient (g/kg)				
Wheat	220.0	216.0	399.0	395.0
Maize	80.0	80.0	-	-
Soybean meal	163.5	163.5	229.2	229.2
Full-fat soybean meal	100.0	100.0	70.0	70.0
Lactofeed 70^2^	200.0	200.0	200.0	200.0
Dried skim milk	125.0	125.0	50.0	50.0
Soybean oil	78.1	78.1	25.0	25.0
Vitamin and mineral premix^3^	3.0	3.0	3.0	3.0
L-Lysine HCl	4.73	4.73	3.70	3.70
DL-Methionine	3.22	3.22	2.33	2.33
L-Threonine	2.41	2.41	1.62	1.62
L-Tryptophan	0.95	0.95	0.54	0.54
Di-calcium phosphate	5.0	5.0	1.52	1.52
Limestone flour	11.0	11.0	11.0	11.0
Salt	3.0	3.0	3.0	3.0
Phytase 5000 FTU/g^4^	0.1	0.1	0.1	0.1
Apralan G200premix^5^	-	1.0	-	1.0
Zinc oxide^6^	-	3.0	-	3.0
Chemical composition (g/kg)				
Dry matter	921	919	889	889
Crude protein	213	212	213	209
Ash	62	64	58	61
Fat	114	114	56	56
Crude fiber	16	19	21	20
Lysine^7^	16.2	16.2	15.0	15.0
Threonine^7^	10.5	10.5	9.8	9.8
Methionine^7^	6.8	6.8	5.7	5.7
Methionine and cysteine^7^	9.7	9.7	9.0	9.0
Tryptophan^7^	3.6	3.6	3.3	3.3
Digestible energy (MJ/kg)^7^	16.2	16.2	15.0	15.0

1 Probiotic treatment was provided by the addition of ∼10^10^ spores/day of *Bacillus pumilus* WIT 588 to the non-medicated treatment.

2 Lactofeed 70 contains 70% lactose, 11.5% protein, 0.5% oil, 7.5% ash, and 0.5% fiber (Volac, Cambridge, UK).

3 Provided the following per kg of complete starter diet: Cu, 155 mg; Fe, 90 mg; Mn, 47 mg; Zn, 120 mg; I, 0.6 mg; Se, 0.3 mg; vitamin A, 6000 IU; vitamin D3, 1000 IU; vitamin E, 100 IU; vitamin K, 4 mg; vitamin B12, 15 µg; vitamin B1, 2 mg; vitamin B6, 3 mg; riboflavin, 2 mg; nicotinic acid, 12 mg; pantothenic acid, 10 mg and choline chloride, 250 mg.

4 Natuphos 5000 (BASF SE, Lampertheim, Germany).

5 Phase 1 and 2 medicated diets contained 200 mg apramycin per kg provided from Apralan G200, (Elanco Animal Health, Eli Lilly & Co., Basingstoke, Hampshire, UK).

6 Phase 1 and 2 medicated diets contained 2500 mg of elemental zinc per kg provided from supplemental zinc oxide (Zincotec; Provimi Ltd., NuTec Mill, Eastern Avenue, Lichfield, Staffordshire, UK) and nutritional zinc included in the vitamin and mineral premix.

7 Calculated values.

### Preparation of *B. pumilus* spores and administration to pigs

*Bacillus pumilus* WIT 588 is a rifampicin resistant variant of a strain (*B. pumilus* WIT 572) previously isolated from seaweed [15], [16]. It was generated to facilitate enumeration in the porcine GIT and characterized *in vitro* as a probiotic for animal production [16]. The strain was previously referred to as *B. pumilus* [15], [16], denoting the fact that it belonged to the *B. pumilus* group, as it could not be distinguished from other members of this group (*B. altitudinis, B. aerophilus, B. safensis, B. stratosphericus*) by 16S rRNA gene sequencing. However, in the present study the identity of the strain was confirmed as *B. pumilus* on the basis of sequencing of the *gyrB* and *pyrE* housekeeping genes [18]. *Bacillus pumilus* WIT 588 was grown aerobically for ∼24 h in Brain Heart Infusion (BHI) broth (Oxoid Ltd, Basingstoke, Hampshire, UK) at 37°C with agitation at 200 rpm. It was then induced to sporulate by spread-plating 1 mL of this culture onto sporulation agar [16], [19] and incubating for 7 d at 37°C. The plates were then flooded with 10 mL sterile ice-cold water and the cells were suspended using a glass spreader. This suspension was heated at 80°C for 15 min to kill any vegetative cells. The spore concentration was determined by diluting the suspension 10-fold in maximum recovery diluent (MRD; Merck, Darmstadt, Germany), and spread-plating on BHI agar incubated at 37°C for 24 h. The concentration was adjusted to ∼10^10^ spores/mL, and aliquots of this spore suspension were stored at −20°C until use.

Aliquots of spore suspension were thawed at 4°C each night before use, as required. On the day of weaning (d 0), pigs on the *B. pumilus* treatment received an oral dose of 5 mL of the spore suspension by syringe immediately after weaning (∼5×10^10^ spores *B. pumilus* WIT 588 per pig). Pigs on the two other treatments received an oral dose of 5 mL sterile distilled water by syringe so that the handling of pigs was identical across treatments. Thereafter (from d 1 to 21), all pigs on the *B. pumilus* treatment received a top dressing of ∼10^10^ spores on their morning feed.

### Animal housing and management

Pigs were housed individually in fully slatted pens (1.2 m×0.9 m) with plastic slats (Faroex, Manitoba, Canada) in a total of four rooms with 12 pigs per room. Each treatment group was represented in each room to avoid possible variation due to environment. The pigs had unlimited access to water from one nipple-in-bowl drinker (BALP, Charleville-Mezieres, Cedex, France) per pen. The temperature was controlled by a hot air heating system and an exhaust fan drawing air from under slat level, both controlled by a Stienen PCS 8400 controller (Stienen BV, Nederweert, The Netherlands). The temperature was maintained at 28-30°C in the first week and reduced by 2°C per week to 24°C. Pigs were observed closely at least three times daily. Any pig showing signs of ill health was treated as appropriate. All veterinary treatments were recorded including identity of pig, symptom, medication used and dosage. Individual body weight, recorded on d 0 and 22 of the study and feed disappearance, recorded on d 0, 8, 15 and 22 of the study, were used for calculation of average daily feed intake (ADFI), average daily gain (ADG), and feed conversion ratio (FCR; ADFI/ADG). Fecal consistency scores (0 =  normal, 1 =  soft, 2 =  mild diarrhea, 3 =  severe, watery diarrhea) [8] were recorded daily during the experiment.

### Blood sampling and analysis

Blood samples were taken from each of 12 pigs/treatment by venipuncture from the anterior vena cava on d 0, 8 and 15. On d 22 of the experiment, 10 pigs/treatment were euthanized by captive bolt stunning followed by exsanguination and blood samples were taken at this time. All samples were collected in plastic blood collection tubes (Vacuette®, Labstock, Dublin, Ireland) and immediately inverted 10 times. Blood samples for serum biochemistry were collected in serum collection tubes, and allowed to clot at room temperature for 2–3 h prior to centrifugation (2000×*g* for 10 min). Serum was collected and stored at −20°C for subsequent biochemical analysis. Whole blood samples were collected in EDTA tubes and stored at room temperature for hematology analysis within 6 h of sampling.

For hematological analysis, the EDTA blood samples were analyzed on a Beckman Coulter Ac T Diff (Beckman Coulter Inc., Brea, CA, USA), as outlined previously [20]. Serum samples were analysed using an ABX Pentra 400 Clinical Chemistry Analyser (Horiba ABX, Northampton, UK), as outlined previously [21].

### Intestinal and organ histology

The kidneys, spleen and liver were removed, trimmed of any superficial fat or blood, blotted dry and weighed (*n* = 10/treatment). Samples of tissue were excised from two anatomical regions of the small intestine: the jejunum (55 cm distal to the pyloric junction) and the ileum (15 cm proximal to the ileo-cecal junction). Samples of liver (centre of quadrate lobe) and kidney (cortex and medulla) were also taken and all samples were immediately placed in No-Tox fixative (Scientific Device Laboratory, Des Plaines, IL, USA) on a shaker for a minimum of 48 h. Intestinal and organ samples were then treated, sliced and mounted as described by Walsh et al. [20] and stained with haematoxylin and eosin (Sigma Aldrich, Ireland) for light microscopic examination. Determination of gross morphological parameters of the structure of the jejunum and ileum (villus height and crypt depth) was conducted according to Applegate et al. [22] and Gao et al. [23]. For each pig 10 villi and 10 crypts were measured on five fields of view, where villi were attached to the lumen. Measurements were taken from images obtained using a light microscope (Olympus, Southend-on-Sea, UK) fitted with an Optikam PRO5 camera (Optika SRL, Ponteranica, Italy) using Optika-Vision Pro software. The goblet cell number was determined in jejunal and ileal sections by periodic acid-Schiff staining according to Thompson & Applegate [24]. Positively stained periodic acid-Schiff cells were enumerated on 10 villi/sample. The means of all parameters were utilized for statistical analysis. All intestinal and organ tissue samples were also examined for histological evidence of abnormality by an experienced histopathologist.

### Fecal and intestinal digesta sampling and microbiological analysis

Fecal samples were obtained by digital rectal stimulation from 12 pigs/treatment on d 0, 8, 15 and 20 and collected in sterile containers. On d 22, digesta samples from the cecum (terminal tip) and ileum (15 cm proximal to the ileo-cecal junction) were collected aseptically into sterile plastic containers. Both digesta and fecal samples were stored at 4°C until analysis (within 12 h). Samples were homogenized as described by Gardiner et al. [25] and 500 µl of each homogenate was heated to 80°C for 15 min. Both heated and unheated homogenates were diluted as described by Gardiner et al. [25]. Appropriate dilutions were plated, as follows; (1) unheated and heated samples were spread-plated on BHI agar containing 200 µg rifampicin/mL, 3.5% NaCl and 50 U/mL nystatin (Sigma) and incubated aerobically for 2 d at 37°C to enumerate the vegetative cells + spores and spores alone, respectively of the administered *B. pumilus* strain; (2) unheated samples were pour-plated on ChromoCult® tryptone bile X- glucuronide (CTBX) agar (Merck) incubated at 44°C for 24 h to enumerate *E. coli*; and (3) unheated samples were pour-plated on *Lactobacillus* selective (LBS) agar (Becton Dickinson, Franklin Lakes, NJ, USA) following anaerobic incubation at 37°C for 5 d to enumerate *Lactobacillus*. Representative putative *E. coli* isolates (four colonies per pig from fecal samples at each time point and ileal and cecal digesta) were streaked onto nutrient agar and then onto CTBX agar to obtain pure cultures. They were then streaked onto eosin methylene blue (EMB) agar (Acumedia Manufacturers, Neogen Europe, Ltd., Auchincruive, Scotland, UK) to confirm identity. To determine if these representative *E. coli* isolates were hemolytic, they were then streaked onto Columbia agar (Sigma) containing 5% sheep blood (TCS Biosciences Ltd., Buckingham, UK).

### Determination of short chain fatty acid concentrations in and pH of ileal and cecal digesta

Samples of cecal and ileal digesta were taken from individual pigs to measure short chain fatty acid (SCFA) concentrations and pH. The pH was measured using a Mettler Toledo pH meter. SCFA concentrations were determined using gas chromatography according to the method of Lynch et al. [26] with the following modifications. A 5 g sample was centrifuged at 1,810×*g* for 10 min, with 1 mL of the resultant supernatant mixed with 1 mL of internal standard. A 1 µL aliquot of centrifuged filtered sample was then injected into an Agilent 5890 gas chromatograph with a 15 m×0.53 mm i.d. Econo-Cap EC-1000 100% polyethylene glycol-acid modified column (Alltech Associates Applied Science Ltd, Carnforth, Lancashire, UK). Nitrogen was used as the carrier gas at a flow rate of 5.6 mL/min. Oven, detector and injector temperatures were set at 82, 280, and 240°C, respectively.

### Statistical Analysis

Data for growth performance, digesta microbiology, histology, organ weights, SCFA concentrations and pH of digesta were analyzed as a complete randomized block design using the mixed models procedure of SAS (SAS Institute, Inc., Cary, NC, USA) [27]. Initial (d 0) body weight and the final (d 22) body weight were used as covariates in the model for analysis of growth performance (BW, ADG, ADFI and FCR) and organ weights, respectively. Fecal microbiology, whole blood hematology and serum biochemistry data were analyzed as repeated measures using the MIXED procedure of SAS with sampling day as the repeated variable. D 0 values were used as a covariate in the model for analysis of hematology, serum gamma glutamyltransferase (GGT) and serum total protein (TP). The appropriate covariance structure, as indicated by the model fit statistics, was fitted to the data. The denominator degrees of freedom were computed using the Satterthwaite approximation. Fixed effects were treatment and sex. Block was included as a random effect. Simple main effects were obtained using the ‘slice’ option in SAS. Least squares means were computed and *P* values were adjusted for multiple comparisons using the Tukey-Kramer adjustment. Significance was reported for *P*≤0.05 and tendencies towards significance were reported for 0.05<*P*≤0.10. For all response criteria, the individual pig was considered the experimental unit.

## Results

### Effects on growth performance and diarrhea scores

On d 12 one pig from the *B. pumilus* group displaying symptoms of pneumonia was treated with injectable enrofloxacin (Baytril, 5% *v/v*, Bayer Ireland, Dublin, Ireland; 1 mL/day) but died on d 13. Also on d 12 one pig from the medicated group displaying symptoms of pneumonia was treated with injectable enrofloxacin (1 mL/day for 3 d) and penicillin (Norocillin, 300 mg/mL, Norbrook, Monaghan, Ireland; 1 mL/day for 3 d). On d 13 another pig from the *B. pumilus* group with symptoms of pneumonia was treated with injectable enrofloxacin and penicillin as before. The latter two pigs made a complete recovery and their data were included in the analysis of growth performance, but excluded from the analysis of all remaining parameters.

The effect of treatment on the growth performance of pigs is presented in Table 2. The initial and final body weight of pigs did not differ between treatments (*P*>0.05), although pigs on the *B. pumilus* treatment tended to be heavier at the end of the experiment than pigs fed the medicated treatment (*P* = 0.07). Although no overall treatment effect was observed for ADFI, there was a tendency for pigs on the *B. pumilus* treatment to have a greater ADFI between d 15 and 22 than pigs fed the medicated treatment (*P* = 0.07). Similarly, a tendency was observed for pigs on the *B. pumilus* treatment to have a higher ADG than pigs fed the medicated treatment (*P* = 0.07). Consequently, pigs on the *B. pumilus* treatment also had improved FCR when compared to pigs on the medicated treatment (*P*<0.05), whereas the FCR of pigs on the non-medicated treatment was similar to that of pigs on the two other treatments.

**Table 2:** Effect of feeding non-medicated, medicated or *B. pumilus* treatments for 22 days on post-weaning pig growth performance.^1^^,^ 2

	Non-medicated	Medicated	*B. pumilus*	SE	*P*
Day 0 BW^3^ (kg)	8.7	8.6	8.8	0.26	0.38
Day 22 BW (kg)	18.1	17.6	18.7	0.35	0.07
ADFI^4^ (g/d)					
Day 0 to 7	182	186	163	10.4	0.12
Day 8 to 14	434	440	446	14.5	0.79
Day 15 to 22	756	713	774	19.8	0.07
Overall	471	458	475	12.6	0.53
ADG^5^ (g/d)	427	405	455	15.7	0.07
FCR^6^	1.11^ab^	1.14^a^	1.05^b^	0.023	0.04

1 Mean values with their standard errors, *n* = 16 for non-medicated and medicated treatments, *n* = 15 for *B. pumilus* treatment.

2 Within each row, values with different superscripts are different at (^a,b^) *P*<0.05.

3 BW  =  body weight.

4 ADFI  =  average daily feed intake.

5 ADG  =  average daily gain.

6 FCR  =  feed conversion ratio (ADFI/ADG).

The mean piglet diarrhea scores for the entire experimental period were 0.34, 0.02 and 0.23 (SE = 0.058; *P*<0.01) for the non-medicated, medicated and *B. pumilus* treatments, respectively (data not shown).

### Effects on hematological parameters

The effect of treatment on hematological parameters is presented in Table 3. No treatment x time interaction or treatment effect was observed for total white blood cell (WBC) counts (*P*>0.05). There was no treatment x time interaction for the lymphocyte percentage (*P*>0.05). Lymphocyte percentage was, however, lower for the *B. pumilus* treatment than for the other two treatments for the overall experimental period (*P*<0.001) and at d 15 (*P*<0.05). At d 8 the lymphocyte percentage was lower for the *B. pumilus* treatment than the medicated treatment (*P*<0.001), with the non-medicated treatment being similar to that of both other treatments (*P*>0.05). There was a treatment x time interaction for monocytes (%) (*P* = 0.01). Monocyte percentage was higher for the non-medicated treatment than for the other two treatments for the overall period (*P*<0.001) and at d 22 (*P*<0.001). At d 8 the percentage of monocytes was higher for the non-medicated treatment than the *B. pumilus* treatment (*P*<0.01), with that of the medicated treatment being similar to both other treatments (*P*>0.05). There was no treatment x time interaction for granulocyte percentage (*P*>0.05). The percentage of granulocytes was higher for the *B. pumilus* treatment than for all other treatments for the overall period (*P*<0.001), at d 8 (*P*<0.001) and at d 15 (*P*<0.01). At d 22, granulocyte percentage was higher for the *B. pumilus* treatment than for the non-medicated treatment (*P*<0.01), with that of the medicated treatment being similar to that of both other treatments (*P*>0.05).

**Table 3:** Effect of feeding a non-medicated, medicated or *B. pumilus* treatment for 22 days post-weaning on hematological parameters of pigs.^1^^,^ 2 ^,^ 3

Day	Treatments			Mean	SE	*P*		
	Non-medicated	Medicated	*B. pumilus*			Treatment	Time	Treatment X Time
Lymphocytes (%)								
8	55.7^ab^	60.0^a^	47.5^b^	54.4	2.32	0.001		
15	66.9^a^	67.1^a^	59.6^b^	64.5	2.54	0.03		
22	77.1	75.4	72.7	75.1	2.41	0.28		
Mean	66.6^a^	67.5^a^	59.9^b^		1.89	<0.0005	<0.0001	0.38
Monocytes (%)								
8	7.7^a^	6.5^ab^	4.5^b^	6.2	0.77	0.004		
15	6.4	6.6	5.0	6.0	0.80	0.26		
22	11.8^a^	7.2^b^	7.0^b^	8.8	0.85	<0.0001		
Mean	8.6^a^	6.8^b^	5.6^b^		0.58	0.0003	<0.0001	0.01
Granulocytes (%)								
8	35.6^b^	33.1^b^	48.1^a^	38.9	2.76	0.0003		
15	25.8^b^	25.8^b^	35.0^a^	28.9	2.58	0.007		
22	10.3^b^	16.9^ab^	20.6^a^	15.9	2.56	0.008		
Mean	24.0^b^	25.3^b^	34.6^a^		1.96	<0.0001	<0.0001	0.20
MCH^4^ (pg)								
8	16.6	16.9	16.9	16.8	0.21	0.58		
15	16.7^ab^	16.3^b^	17.4^a^	16.7	0.20	0.03		
22	17.7^a^	16.3^b^	17.5^a^	17.2	0.22	<0.0001		
Mean	17.0^ab^	16.5^b^	17.2^a^		0.19	0.03	<0.0001	<0.0001

1 Mean values with their standards errors, *n* = 12 on d 0, 8 and 15, *n* = 10 on d 22.

2 Within each row, values with different superscripts are different at (^a,b^) *P*<0.05.

3 D 0 values were used as covariate in the statistical model.

4 MCH, mean corpuscular hemoglobin.

There was a treatment x time interaction for the erythrocyte index, mean corpuscular hemoglobin (MCH) (*P*<0.001). The MCH was also lower for the medicated treatment compared to the *B. pumilus* treatment for the overall experimental period (*P*<0.05) and at d 15 (*P*<0.05), with that for the non-medicated treatment being similar to that of both other treatments for the overall period (*P*>0.05). At d 22, MCH was higher for the *B. pumilus* and non-medicated treatments than the medicated treatment (*P*<0.001), with no difference observed between the former two treatments (*P*>0.05). There was a tendency for a treatment x time interaction for the erythrocyte index, mean corpuscular volume (MCV) (*P* = 0.07), but no treatment effect was observed for the overall period (*P*>0.05). At d 15 there was a tendency for MCV to be lower in pigs on the medicated treatment than for pigs on the two other treatments (*P* = 0.08). There was a treatment x time interaction for the erythrocyte index, mean corpuscular hemoglobin concentration (MCHC) (*P*<0.001) and although there was no overall treatment effect (*P*>0.05), MCHC tended to be higher at d 8 for the *B. pumilus* treatment than the non-medicated treatment (*P* = 0.08). There was no treatment x time interaction or treatment effect for the erythrocyte index, red blood cell distribution width (RDW) (*P*>0.05). Nevertheless, at d 22 RDW was 24.9, 27.7 and 23.0% (SE = 1.25%; *P*<0.05) for the non-medicated, medicated and *B. pumilus* treatments, respectively (data not shown). For the remainder of the hematological parameters measured [red blood cell (RBC) counts, hemoglobin, hematocrit, mean platelet volume (MPV) and platelet counts] no treatment x time interaction or treatment effects were observed (*P*>0.05; data not shown).

### Effects on serum biochemistry

The effect of treatment on serum biochemistry is presented in Table 4. There was no treatment x time or overall treatment effect on creatinine concentration (*P*>0.05). However, at d 15 the creatinine concentration was 77.5, 83.1 and 76.9 µM/L (SE = 1.93 µM/L; *P*<0.05) for the non-medicated, medicated and *B. pumilus* treatments, respectively (data not shown). There was a treatment x time interaction for urea concentration (*P*<0.001) but no overall treatment effect (*P*>0.05). At d 8, urea concentration was 3.1, 1.9 and 3.0 mM (SE = 0.29 mM/L; *P*<0.01) for the non-medicated, medicated and *B. pumilus* treatments, respectively (data not shown). At d 22 urea concentration was 3.9, 4.4 and 3.6 mM (SE = 0.22 mM/L; *P*<0.05) for the non-medicated, medicated and *B. pumilus* treatments, respectively (data not shown).

**Table 4:** Effect of feeding a non-medicated, medicated or *B. pumilus* treatment for 22 days post-weaning on serum biochemistry parameters of pigs.^1^^,^ 2

Day	Treatments			Mean	SE	*P*		
	Non-medicated	Medicated	*B. pumilus*			Treatment	Time	Treatment X Time
ALT (alanine aminotransferase) (U/L)								
8	23.7	25.4	21.7	23.6	1.38	0.12		
15	33.8^b^	39.6^a^	32.0^b^	35.1	1.77	0.01		
22	40.5^b^	69.4^a^	41.5^b^	50.6	7.40	0.01		
Mean	34.1^b^	44.4^a^	33.6^b^		2.30	0.002	<0.0001	0.10
ALP (alkaline phosphatase) (U/L)								
8	308^b^	498^a^	339^b^	382	28.8	<0.0001		
15	347^b^	686^a^	407^b^	480	33.2	<0.0001		
22	333^b^	846^a^	418^b^	532	51.8	<0.0001		
Mean	372^b^	624^a^	422^b^		28.3	<0.0001	<0.0001	<0.0001
GGT^3^ (gamma glutamyltransferase) (U/L)								
8	35.1	39.3	38.4	37.6	1.95	0.32		
15	38.6^b^	43.8^a^	38.3^b^	40.2	1.37	0.003		
22	36.2^b^	50.0^a^	39.7^b^	35.1	2.37	0.001		
Mean	36.7^b^	44.4^a^	38.8^b^		1.62	0.002	0.01	0.02

1 Mean values with their standard errors, *n* = 12 on d 0, 8 and 15, *n* = 10 on d 22.

2 Within each row, values with different superscripts are different at (^a,b^) *P*<0.05.

3 GGT d 0 values were used as covariate in the statistical model, as there were significant differences.

There was a tendency for a treatment x time interaction for serum alanine aminotransferase (ALT) concentration (*P* = 0.10) and there was an overall treatment effect (*P*<0.01). Serum ALT concentration was increased in the medicated compared with the non-medicated and *B. pumilus* treatments for the overall period (*P*<0.01), as well as at d 15 (*P*<0.01) and d 22 (*P*<0.01). There was a treatment x time interaction (*P*<0.001) for serum alkaline phosphatase (ALP) concentration. Serum ALP was higher for the medicated than the non-medicated and *B. pumilus* treatments for the overall period (*P*<0.001) and at d 8 (*P*<0.001), d 15 (*P*<0.001) and d 22 (*P*<0.001). There was a treatment x time interaction for serum GGT concentration (*P*<0.05). Serum GGT concentration was higher for the medicated treatment than for all other treatments for the overall period (*P*<0.01) and at d 15 (*P*<0.01) and d 22 (*P*<0.001). There was no treatment x time interaction or treatment effect for serum aspartate aminotransferase (AST). There was a treatment x time interaction for serum TP (*P*<0.05) but no overall treatment effect (*P*>0.05).

### Effects on organ weights and histology

The effect of treatment on organ weights is shown in Table 5. Treatment did not affect renal or splenic weight; however, pigs on the medicated treatment had lighter livers than pigs on either the non-medicated or *B. pumilus* treatments (*P*<0.05). Histopathological examination of the kidneys did not reveal any abnormalities for pigs on any of the treatments. Subtle inflammation was observed in the liver of one pig on the medicated treatment and in one pig on the *B. pumilus* treatment, but in both cases the inflammation was classified as ‘very mild’ in character and was most likely subclinical.

**Table 5:** Effect of feeding a non-medicated, medicated or *B. pumilus* treatment for 22 days post-weaning on organ weights (g) of pigs.^1^^,^ 2 ^,^ 3

	Non-medicated	Medicated	*B. pumilus*	SE	*P*
Kidneys	110.7	114.8	107.0	3.74	0.23
Spleen	38.6	37.4	33.8	2.04	0.17
Liver	554.5^a^	503.5^b^	547.9^a^	15.46	0.02

1 Mean values with their standard errors, *n = *10.

2 Organ weights were analyzed using the final body weight on d 22 as a covariate.

3 Within each row, values with different superscripts are different at (^a,b^) *P*<0.05.

### Effects on small intestinal histology

The effect of treatment on small intestinal histology is shown in Table 6. There was a tendency for jejunal villus height to be higher for the *B. pumilus* and the medicated treatments compared to the non-medicated treatment (*P* = 0.10). Treatment had no effect on crypt depth, villus width or villus height:crypt depth ratio in the jejunum (Table 4). The number of goblet cells/villus in the jejunum was higher for the *B. pumilus* and the medicated treatments compared to the non-medicated treatment (*P*<0.01); however, the number of goblet cells/µm of villus was not affected by treatment (*P*>0.05). Treatment had no effect on any of the histological parameters investigated in the ileum. Histopathological examination revealed crypt inflammation in the ileum of one pig on the *B. pumilus* treatment, but this was categorized as very subtle and unlikely to be of clinical significance.

**Table 6:** Effect of feeding a non-medicated, medicated or *B. pumilus* treatment for 22 days post-weaning on small intestinal histology of pigs.^1^^,^ 2 ^,^ 3

	Non-medicated	Medicated	*B. pumilus*	SE	*P*
Jejunum					
Villus height (µm)	422	471	497	25.5	0.10
Crypt depth (µm)	305	322	340	17.0	0.34
Villus width (µm)	185	187	193	15.5	0.94
Villus height: crypt depth ratio	1.41	1.54	1.51	0.127	0.73
Number of goblet cells/villus	9.67^a^	14.30^b^	13.97^b^	0.963	0.002
Number of goblet cells/µm of villus	0.025	0.031	0.029	0.0019	0.13
Ileum					
Villus heights (µm)	353	332	355	16.5	0.49
Crypt depth (µm)	183	191	202	13.6	0.56
Villus width (µm)	158	159	151	5.0	0.54
Villus height: crypt depth ratio	2.02	1.94	1.79	0.176	0.58
Number of goblet cells/villus	12.66	13.58	12.82	0.874	0.72
Number of goblet cells/µm of villus	0.037	0.042	0.038	0.0027	0.36

1 Mean values with their standard errors, *n* = 10.

2 Ten villi and 10 crypts were measured on five fields of view for each pig and the means were utilized for statistical analysis.

3 Within each row, values with different superscripts are different at (^a,b^) *P*<0.05.

### Fecal shedding and intestinal survival of the administered *B. pumilus* strain and effects on fecal and intestinal *E. coli* and *Lactobacillus*

Data on fecal shedding of the administered *B. pumilus* strain and the effect of treatment on selected culturable fecal microbiota are presented in Table 7. There was a treatment x time interaction for *E. coli* counts (*P*<0.01). *E. coli* counts were lower for the medicated than both the non-medicated and *B. pumilus* treatments for the overall period (*P*<0.01) as well as at d 8 (*P*<0.001) and d 15 (*P*<0.01). However, none of the representative fecal *E. coli* isolates examined were hemolytic. There was no treatment x time interaction for fecal *Lactobacillus* counts (*P*>0.05). *Lactobacillus* counts were lower for the medicated than both the non-medicated and *B. pumilus* treatments for the overall period (*P*<0.001) and at d 8 (*P*<0.01). At d 15 and d 20, *Lactobacillus* counts were lower for the medicated than the *B. pumilus* treatment (*P*<0.01) while *Lactobacillus* counts for the non-medicated treatment were similar to those of both other treatments (*P*>0.05). The administered *B. pumilus* strain (vegetative cells plus spores as well as spores alone) was detected in the feces of all pigs on the *B. pumilus* treatment at all time points except d 0. The administered strain was not recovered from any of the pigs on either the non-medicated or medicated treatments throughout the experiment.

**Table 7:** Effect of feeding a non-medicated, medicated or *B. pumilus* treatment for 22 days post-weaning on fecal bacterial counts (log_10_ CFU/g) of pigs and fecal counts of the administered *B. pumilus* strain.^1^^,^ 2 ^,^ 3

Day	Treatments			Mean	SE	*P*		
	Non-medicated	Medicated	*B. pumilus*			Treatment	Time	Treatment X Time
*E. coli*								
0	8.24	8.10	7.37	7.90	0.490	0.36		
8	6.84^a^	4.12^b^	6.63^a^	5.86	0.400	<0.0001		
15	6.05^a^	4.35^b^	6.10^a^	5.50	0.400	0.003		
20	5.71	4.98	5.49	5.39	0.400	0.41		
Mean	6.71^a^	5.39^b^	6.40^a^		0.250	0.002	<0.0001	0.003
*Lactobacillus*								
0	8.17	7.88	8.02	8.03	0.320	0.84		
8	9.59^a^	8.44^b^	9.45^a^	9.16	0.215	0.001		
15	9.2^ab^	8.68^b^	9.60^a^	9.16	0.194	0.008		
20	9.07^ab^	8.68^b^	9.27^a^	9.01	0.123	0.006		
Mean	9.01^a^	8.42^b^	9.08^a^		0.069	<0.0001	<0.0001	0.27
*B. pumilus* WIT 588 (vegetative cells + spores)								
0	ND^4^	ND	ND					
8	ND	ND	6.22					
15	ND	ND	5.99					
20	ND	ND	5.80					
Mean	ND	ND	5.06					
*B. pumilus* WIT 588 (spores)								
0	ND	ND	ND					
8	ND	ND	6.10					
15	ND	ND	5.70					
20	ND	ND	5.61					
Mean	ND	ND	4.86					

1 Mean values with their standard errors, *n* = 12.

2 Bacterial counts are presented as log_10_ CFU/g wet weight.

3 Within each row, values with different superscripts are different at (^a,b^) *P*<0.05.

4 Non-detectable (the limit of detection was 100 CFU/g i.e. log_10_ 2.0 CFU/g), although at d 0 low values were recorded for the vegetative cells + spores count in some pigs, representing background rifampicin resistant microflora.

Data on ileal and cecal counts of the administered *B. pumilus* strain and the effect of treatment on selected culturable ileal and cecal microbiota are presented in Table 8. *E. coli* counts in the ileum were lower for the medicated and *B. pumilus* treatments compared to the non-medicated treatment (*P*≤0.05), but no treatment effect was observed for cecal *E. coli* counts (*P*>0.05). However, none of the representative *E. coli* isolates recovered from either the ileum or cecum were hemolytic. *Lactobacillus* counts in the ileum were not affected by treatment (*P*>0.05), while cecal *Lactobacillus* counts were lower for the medicated than the non-medicated and *B. pumilus* treatments (*P*<0.05). The administered *B. pumilus* strain (vegetative cells plus spores as well as spores alone) was detected in both the ileum and cecum of all pigs on the *B. pumilus* treatment but not from pigs on either the non-medicated or medicated treatments.

**Table 8:** Effect of feeding a non-medicated, medicated or *B. pumilus* treatment for 22 days post-weaning on ileal and cecal bacterial counts (log_10_ CFU/g) of pigs and ileal and cecal counts of the administered *B. pumilus* strain.^1^^,^ 2 ^,^ 3

	Non-medicated	Medicated	*B. pumilus*	SE	*P*
Ileum					
* E. coli*	4.01^a^	2.61^b^	2.88^b^	0.394	0.05
* Lactobacillus*	8.75	8.63	8.40	0.154	0.28
* B. pumilus* WIT 588 (vegetative cells + spores)	ND^4^	ND	2.61		
* B. pumilus* WIT 588 (spores)	ND	ND	2.73		
Cecum					
* E. coli*	4.49	4.68	5.44	0.395	0.34
* Lactobacillus*	9.10^a^	8.66^b^	9.07^a^	0.116	0.02
* B. pumilus* WIT 588 (vegetative cells + spores)	ND	ND	3.70		
* B. pumilus* WIT 588 (spores)	ND	ND	3.92		

1 Mean values with their standard errors, *n* = 10.

2 Bacterial counts are presented as log_10_ CFU/g^−1^ wet weight.

3 Within each row, values with different superscripts are different at (^a,b^) *P*<0.05.

4 Non-detectable (the limit of detection was 100 CFU/g i.e. log_10_ 2.0 CFU/g).

### Effects on ileal and cecal SCFA concentrations and pH

The effect of treatment on SCFA concentrations in the ileum and cecum and on the pH of the ileal and cecal digesta are presented in Table 9. The pH of the ileal digesta was higher for pigs on the *B. pumilus* treatment compared to those on the non-medicated treatment (*P*≤0.05), with pigs on both treatments having ileal digesta with a similar pH to those on the medicated treatment (*P*>0.05). The pH of the cecal digesta was higher for pigs on the medicated treatment compared to those on the non-medicated and *B. pumilus* treatments (*P*<0.01) with the latter two treatments having cecal content with a similar pH (*P*>0.05).

**Table 9:** Effect of feeding a non-medicated, medicated or *B. pumilus* treatment for 22 days post-weaning on pH of and short chain fatty acid concentrations (mM/g) in ileal and cecal digesta of pigs.^1^^,^ 2

	Non-medicated	Medicated	*B. pumilus*	SE	*P*
Ileum					
pH	6.73^b^	7.21^ab^	7.34^a^	0.168	0.05
Acetic acid	3.16^b^	4.77^a^	3.51^ab^	0.435	0.04
Propionic acid	0.46^b^	1.61^a^	1.7^a^	0.213	0.001
Isobututyric acid	0.06	0.02	0.05	0.029	0.43
Butyric acid	0.57	1.02	1.15	0.183	0.08
Isovaleric acid	0.44	0.31	0.30	0.097	0.52
Valeric acid	0.3	0.19	0.23	0.116	0.79
Total	5.01^b^	7.92^a^	6.94^ab^	0.672	0.01
Cecum					
pH	5.79^b^	6.32^a^	5.84^b^	0.117	0.006
Acetic acid	37.65	33.48	43.76	3.061	0.08
Propionic acid	22.7^a^	17.13^b^	22.18^a^	1.686	0.007
Isobututyric acid	0.14	0.21	0.23	0.040	0.25
Butyric acid	11.49	8.55	9.71	1.520	0.38
Isovaleric acid	0.57	0.63	0.44	0.064	0.10
Valeric acid	2.05^a^	0.91^b^	1.32^ab^	0.272	0.02
Total	74.85	61.15	77.36	5.540	0.10

1 Mean values with their standard errors, *n* = 10.

2 Within each row, values with different superscripts are different at (^a,b^) *P*<0.05.

The total concentration of SCFA in the ileum was higher for the medicated treatment than for the non-medicated treatment (*P*<0.01) with that for the *B. pumilus* treatment being similar to that of both other treatments (*P*>0.05). This was also the case for acetic acid concentrations in the ileum (*P*<0.05). The ileal concentration of propionic acid was similar for the medicated and *B. pumilus* treatments, but both treatments had higher concentrations than that found in the non-medicated treatment (*P*<0.001). There was a tendency for butyric acid concentration in the ileum to be higher for both the medicated and *B. pumilus* treatments than for the non-medicated treatment (*P* = 0.08). Total SCFA concentrations in the cecum tended to be higher for the non-medicated and *B. pumilus* treatments than for the medicated treatment (*P* = 0.10). This pattern was significant for propionic acid concentrations in the cecum (*P*<0.01). Higher cecal valeric acid concentrations were found for the non-medicated treatment compared to the medicated treatment (*P*<0.05), while both treatments had similar concentrations to those of the *B. pumilus* treatment (*P*>0.05). There was a tendency for cecal acetic acid concentrations to be higher in pigs on the *B. pumilus* treatment than the other two treatments (*P* = 0.08). Pigs on the medicated treatment tended to have a higher concentration of isovaleric acid in the cecum than those on the *B. pumilus* treatment (*P* = 0.10).

## Discussion

The present study evaluates, for the first time, the safety and efficacy of a marine-derived *Bacillus* strain for use as an in-feed probiotic in newly weaned pigs. The *B. pumilus* used was pre-screened and found to satisfy a number of probiotic criteria and was selected in particular for its ability to inhibit porcine pathogenic *E. coli* [16].

A number of studies to date have evaluated various *Bacillus* strains for use as probiotics for pigs [28]. However, one of the novel aspects of the present study is that intestinal survival of the administered strain was proven via use of an antibiotic-marked strain, a strategy that we have previously used in other studies [25]. Fecal shedding was relatively high, compared to intestinal recovery. This, together with the fact that practically the entire count recovered from both the feces and the intestine can be accounted for by spores, indicates that the *Bacillus* spores may be merely transiting the GIT. This is despite the fact that the *B. pumilus* strain grows and its spores have been shown to germinate under anaerobic conditions *in vitro* [16]. Nonetheless, a number of beneficial effects were observed. For example, *B. pumilus* administration resulted in reduced ileal *E. coli* counts and an ∼8% improvement in FCR compared to the medicated treatment. The latter resulted principally due to a 12% increase in ADG, although this was only a tendency.

However, it should be borne in mind that these improvements in growth performance were only observed in comparison to the medicated feed containing a combination of apramycin and pharmacological levels of zinc oxide. In fact, the data indicate poorer ADG and FCR as a result of in-feed medication. Pharmacological levels of zinc oxide are commonly added to pig diets to prevent post-weaning diarrhea and improve growth performance [1]. Although its mechanism of action is unclear [1], zinc, at high concentrations, is thought to be active against Gram-negative bacteria, such as *E. coli*, even though *in vivo* data do not back this up [29]–[31]. Apramycin is an antibiotic that is mainly used to treat *E. coli* infections, such as porcine colibacillosis [32] and it has previously been shown to improve growth performance in weaned pigs [32], [33], although performance effects can vary depending on age, health status and environment. Interestingly, in the present study the medicated treatment did reduce the diarrhea score, as well as fecal and ileal *E. coli* counts. The latter can be considered beneficial, given that increased *E. coli* counts are associated with a decline in growth and increased diarrhea in pigs, especially when subject to stressors, such as weaning [34]. However, none of the representative fecal or intestinal *E. coli* examined were hemolytic, indicating that they may not have been pathogenic and may, in fact, be harmless, or even beneficial commensals. Although only a representative number of *E. coli* isolates were examined, the fact that none appear pathogenic explains the fact that edema or diarrhea were not observed during the experiment, even though there was a history of post-weaning edema disease within the herd. The medicated treatment also lowered counts of fecal and cecal *Lactobacillus* and this would not be considered favorable, given that this genus is thought to have a beneficial role within the porcine GIT [35]. Others have reported *Lactobacillus* reductions in the stomach, ileum, cecum and colon digesta and decreases in lactic acid bacteria in the colonic mucosa in weaned pigs administered pharmacological levels of zinc oxide or antibiotics, respectively in the diet [13], [31]. Reduced *Lactobacillus* counts, together with the reduction in possible *E. coli* commensals may explain the negative growth performance effects observed in pigs administered the medicated treatment.

Interestingly, the *B. pumilus* strain was as effective as in-feed medication (apramycin plus zinc oxide) in reducing ileal *E. coli* counts, although it had no effect on *E. coli* shedding in the feces. However, *E. coli* inhibition in the ileum is probably more relevant as regards disease prevention; for example, lowering ileal *E. coli* has been proposed as one of the means by which edema disease can be prevented in weaned pigs [36]. Previous studies with *Bacillus* probiotics have mainly shown reductions in fecal shedding of *E. coli* [9]–[12], but one in which pigs were challenged with a serotype of *E. coli* known to cause edema disease, also demonstrated reductions in the ileal digesta [36]. Furthermore, the decrease in *E. coli* observed in the present study was achieved without the reductions in potentially beneficial *Lactobacillus* that occurred with the medicated feed. This is explained by the fact that the *B. pumilus* strain inhibited porcine *E. coli in vitro* but had only limited anti-*Lactobacillus* activity [16]. However, it should be noted, as outlined above, that none of the representative intestinal *E. coli* isolates examined in the present study appeared pathogenic.

Apart from the fact that the *B. pumilus* strain administered in the present study has antimicrobial activity against *E. coli in vitro* [15], [16], the reductions in ileal *E. coli* observed *in vivo* could potentially be explained by the higher concentrations of propionic acid in the ileum of these pigs compared with those fed the non-medicated treatment. This is because propionic acid has been suggested to reduce the growth of *Enterobacteriaceae* [37]. Intestinal concentrations of SCFA are often increased by *Bacillus* administration [38] and this is likely to be as a result of carbohydrate degradation by the administered strains [39]. The reduction in ileal *E. coli* counts could also lead to the proliferation of endogenous bacteria and this could be responsible for the increased propionic acid concentrations. In contrast, the tendency for lower concentrations of cecal SCFA observed in pigs fed the medicated treatment could be associated with the lower counts of cecal lactobacilli in these animals. However, a global approach using non-culture-based methods would be required to thoroughly examine the effects on the intestinal microflora and such an approach would help to explain these phenomena.

The *B. pumilus* administered in the present study lowered *E. coli* counts without toxic effects. In contrast, liver weight was ∼10% less for pigs fed the medicated treatment compared to that of pigs fed either the non-medicated or *B. pumilus* treatment, which could be the result of chronic toxicity of apramycin, as previously reported by the manufacturers [40]. The liver can atrophy, leading to a reduction in size, when it is unable to regenerate following a period of ongoing insult [41]. Apramycin could have toxic effects, as, although it is excreted by the kidneys as the intact molecule [42], high concentrations can accumulate in kidney and liver tissue [43]. There are also suggestions that administration of elevated levels of zinc oxide to pigs post-weaning may have toxic effects, although this is largely unsubstantiated. While our study found no evidence of histopathological abnormalities in the liver, overall serum concentrations of the liver enzymes, GGT, ALT and ALP were 21, 30.2 and 67.7% higher, respectively, in pigs fed the medicated treatment, indicating possible liver damage [44]–[46]. However, despite the differences found, serum GGT and ALT concentrations were within normal ranges for pigs and only the serum ALP concentration of pigs on the medicated treatment was higher than the normal range [41]. The higher concentrations of serum creatinine found in pigs fed the medicated treatment could indicate kidney damage; however, these differences were only observed at one time point and the latter was only compared to the *B. pumilus* treatment. Furthermore, kidney damage is also normally associated with changes in kidney weight and reduced serum protein concentrations [46], [47], neither of which were found in the present study. In addition, the overall concentrations of serum creatinine and urea were inside the normal ranges for pigs of this age [46].

Pigs on the *B. pumilus* treatment had lower percentages of lymphocytes and monocytes, compared to pigs on the non-medicated and medicated treatments, and to pigs on the non-medicated treatment, respectively. However, this would not necessarily be a cause for concern, as it is the opposite (i.e. elevated concentrations of these blood cells) that is undesirable due to its association with infection [48]. Furthermore, counts remained within the normal ranges for pigs throughout the experiment [41], [48]. However, we did observe a higher percentage of granulocytes in pigs administered the *B. pumilus* strain and this is characteristic of an inflammatory response [48], but again the counts remained within normal ranges [41], [48]. The increased density of goblet cells in the jejunal epithelium of pigs on the *B. pumilus* and the medicated treatments may also be indicative of an inflammatory response, as has previously been observed in weaned pigs in response to total parenteral nutrition [49]; however, histopathological examination revealed no signs of intestinal inflammation except for subtle crypt changes in the ileum of one pig which was considered unlikely to be of clinical significance. There were also some differences in two of the RBC indices in pigs fed the *B. pumilus* treatment i.e. higher MCH content (lower hemoglobin in RBC) and lower RDW. However, these were only observed in comparison to the medicated treatment. Most of the RBC and platelet indices were slightly lower than the normal ranges for 36-d old pigs [48]. Low hematocrit, RBC count and hemoglobin concentrations could be an indication of anemia [41]; however, supplemental iron was administered to all pigs prior to the experiment at 3 days post-partum. The lower than normal range values were found across all treatments and could be explained by factors such as breed and management practices [48], [50].

## Conclusions

The data from the present study indicate that dietary supplementation with a marine-derived *B. pumilus* strain improved the growth performance of newly weaned pigs but only when compared to a medicated treatment containing apramycin and zinc oxide. The *B. pumilus* treatment decreased ileal *E. coli* counts in a manner similar to the medicated treatment but without the reduction in growth performance, decreased fecal and cecal *Lactobacillus* counts, reduction in cecal SCFA and possible liver toxicity found with the medicated treatment. The higher granulocyte percentage and increased jejunal goblet cells found in pigs on the *B. pumilus* treatment may be indicative of a response to inflammation/local injury. However, no other intestinal or organ abnormalities were observed and all serum biochemistry and most hematological parameters were within normal ranges. Overall, these data indicate that the marine *B. pumilus* strain tested in the present study appears safe for use as a probiotic in weaned pigs and demonstrates potential for use as an alternative to in-feed antibiotics. However, additional investigations, including culture-independent analysis of the intestinal microflora and more detailed immunological assays are warranted.
